# Protective Actions of Acidic Hydrolysates of Polysaccharide Extracted From *Mactra veneriformis* Against Chemical-Induced Acute Liver Damage

**DOI:** 10.3389/fphar.2020.00446

**Published:** 2020-04-24

**Authors:** Lingchong Wang, Ying Yang, Hor-Yue Tan, Sha Li, Yibin Feng

**Affiliations:** ^1^ School of Pharmacy, Nanjing University of Chinese Medicine, Nanjing City, China; ^2^ School of Chinese Medicine, LKS Faculty of Medicine, The University of Hong Kong, Hong Kong, Hong Kong

**Keywords:** *Mactra veneriformis*, hydrolysates, polysaccharide, acute liver damage, liver-protection agent

## Abstract

The present study aimed to explore the hepatoprotective effects of acidic hydrolysates of polysaccharide extracted from the marine clam *M. veneriformis* (Ah-MVPS) against ethanol- and CCl_4_-induced liver damage. Moreover, we also seek to probe the mechanism associated with the liver protection effect of Ah-MVPS. A series of animal and cell experiments were executed to detect suitable serological and histological indicators in hepatic tissues. Ah-MVPS can significantly reduce liver damage by means of an increase in hepatocyte superoxidase dismutase and inhibition of leakages of alanine aminotransferase and aspartate transaminase, as well as through alleviation of malondialdehyde excalation. Ah-MVPS inhibited steatosis and water-like hepatic deterioration in histological examination. They can suppress membrane destruction in boundaries and the collapse of reticular scaffolds of injured mouse hepatocytes and can substantially reduce the inflammatory extent of liver tissue aroused by excessive intake of ethanol or CCl_4_. In cell assays, Ah-MVPS markedly elevated the viability of L-02 cells exposed to an intoxication of ethanol or H_2_O_2_. The beneficial effect of Ah-MVPS might arise, at least in part, because of the amelioration of peroxidation or oxidative stress. Taken together, our findings reveal that Ah-MVPS have potential for development as protective agents to attenuate acute liver injuries.

## Introduction

As the largest digestive gland, the liver has the capacity to defend against chemical jeopardy *via* decomposition or metabolism of exogenous or endogenous toxins that damage the health of the human body (Yang et al., 2013; Uyanoglu et al., 2014). However, the liver itself may suffer from such harmful attacks from, e.g., ethanol, virus, drugs, out-of-control immune response, and hypoxia (Xiao et al., 2018). Damage to the liver involves many pathological changes, such as hepatocyte inflammation, steatosis, fibrosis, and necrosis, which can be broadly divided into infection and chemical injuries. Chemical liver injury is usually defined as chemical-driven hepatocyte damage accompanying acute inflammation, since the hepatocyte is susceptible to many absorbable chemical toxins, and the severity of the pathology is strongly related to type, dose, and period of chemical intakes (Gu and Manautou, 2012). Chemical liver injuries easily progress to severe terminal liver disease, such as cirrhosis and hepatocarcinogenesis, by combination with other causes or through repeated episodes. It is essential to find or screen out some compounds that are protective to liver, given the recognized urgency of prevention and treatment of liver diseases. Animals with liver injury induced by CCl_4_ or ethanol are typical experimental models that have been comprehensively applied in efficacy evaluation and pharmacological investigation of liver-protective drugs, as well as for screening of active compounds (Sun et al., 2013).

Reactive oxygen species (ROS) play an important role in the initiation and progression of chemical liver damage (Poli, 1993; Muriel, 2009). Excessive ROS generation, inducing imbalance of the intracellular oxidative-antioxidant system, is perhaps the chief pathological mechanism of chemical liver injury. Specifically, long-term or high doses of chemical intake and subsequent metabolism will produce multitudinous ROS beyond the elimination ability of the hepatic antioxidant enzymes, such as superoxidase dismutase (SOD), and will finally disturb the cellular homeostasis and lead to accumulation of liver damage (Pushpavalli et al., 2010). Excess free radicals bind to important biomolecules on the surfaces of mitochondria or endoplasmic reticulum, leading to lipid peroxidation and producing malondialdehyde (MDA). This will cause endoplasmic reticulum stress (ERS) and mitochondrial outer membrane permeabilization (MOMP), which are characterized by a decrease in fluidity and an increase in permeability of the membrane, release of cytochrome C, activation of capsase, and eventual cell apoptosis or necrosis (Liu et al., 2018).

It has been widely confirmed by the contemporary academic community that natural polysaccharides and/or their oligomer derivatives are free radical scavengers that are successfully applied in Chinese medicine or alternative medicine as protective agents to prevent liver damage (Tong et al., 2015; Xiao et al., 2018). Numerous natural polysaccharides/oligosaccharides can reduce consumption of the antioxidant factors that are induced by an increase in oxidative stress, thus inhibiting the oxidative degeneration of lipids, enzymes, and nucleic acids and ultimately protecting the cell membrane structure and safeguarding the organelles (Wang et al., 2014; Wang et al., 2019).

*Mactra veneriformis,* sometimes termed *M. quadrangularis,* is an edible seashore clam (shellfish) that has been widely cultured and largely consumed as a seafood resource in many Asian countries (http://www.sealifebase.org/summary/Mactra-quadrangularis.html). Its fleshy part is also historically used as traditional Chinese Medicine (TCM) to remedy liver function injured by alcohol abuse (http://zhongyaocai360.com/g/geli.html). The hepatoprotective effect of *M. veneriformis* and the related active ingredients has been primarily investigated by our research group (Luan et al., 2011; Wang et al., 2011a). The first result obtained was that the extracted polysaccharide (MVPS) could provide hepatoprotective benefits for CCl_4_-induced liver-damaged mice. One derivative, hydrolysates of MVPS from HCl reaction, were then verified to have more superior antioxidant activity in comparison with the maternal polysaccharide (Wang et al., 2016). It was finally acknowledged that HCl hydrolysis might modify the physicochemical characters of the polysaccharide and improve its biological activity. In brief, the hydrolyzing products of MVPS varied in compositions and oligosaccharide contents and performed significant antioxidant actions by scavenging DPPH radicals, inhibiting the hydroxyl radicals, and generating reducing power. This indicated that some oligosaccharides might be functional ingredients since the hydrolysate exhibited stronger antioxidant effect than the initial polysaccharide of MVPS.

The above beneficial effect of MVPS and its hydrolysis products on mouse liver-injury or radical scavenging shifted our attention in subsequent studies toward the *in vivo* antioxidant mechanism and liver-protective actions. In the current study, we investigated the potential protective effects of the acidic hydrolysates of *M. veneriformis* polysaccharides (Ah-MVPS) in mitigating ethanol- or CCl_4_-elicited liver injuries. Moreover, at the cellular level, the liver protective effect was further verified. Our findings demonstrate that Ah-MVPS have promising potentials for development as protective agents for patients who are susceptible to liver diseases.

## Materials and method

### Materials

Three-year-old clams were collected by hand picking from the sea beach of Lvsi harbor at Nantong, Jiangsu province, China (32°06 N; 122°30 E) and were identified as *M. veneriformis* by Jiangsu Marine Fisheries Research Institute. The captured clams were starved in an aquarium for 24 h to evacuate their gut contents, and then their flesh was excavated from their shells and stored at −10 °C for further use.

Dulbecco’s modified Eagle medium (DMEM) and Dulbecco’s phosphate-buffered saline (DPBS) was obtained from Invitrogen. Fetal bovine serum (FBS) was acquired from Gibco. Phenazine methosulfate (PMS) and ethylenediaminetetraacetic acid (EDTA) were purchased from Amresco. A methanethiosulfonate (MTS) kit was purchased from Promega. Pancreatin was obtained from Beijing Dingguo Biological Reagent Co., Ltd. Carbon tetrachloride (CCl_4_) was purchased from Sinopharm Chemical Reagent Co., Ltd. Bifendatatum (Biphenyl diester dropping pills) was provided by Beijing Union Pharmaceutical Factory. Standard commercial bioassay kits for alanine aminotransferase (ALT), aspartate transaminase (AST), superoxidase dismutase (SOD) malondialdehyde (MDA), and protein BCA determinations were purchased from Nanjing Jiancheng Bioengineering Institute. All other reagents and che micals employed in the present work were analytical grade and supplied by local chemical suppliers in Nanjing city.

### Preparation of Ah-MVPS

Water-soluble polysaccharide from the clam, utilized as the initial material in this study, was prepared by hot-water extraction and alcoholic precipitation, as described in our previous publication (Wang et al., 2011b). Briefly, pre-washed flesh materials of *M. veneriformis* were cut into pieces by a mincer and then decocted for 2 h in six-fold volumes of boiling water. The decoctions were centrifuged at 6000 rpm for 20 min, and the supernatant was concentrated to double volume and then precipitated by the addition of quadruple volumes of alcohol at room temperature. After overnight reaction, the precipitates were collected by filtration with 400-mesh fabrics. The products were dehydrated by 95% ethanol and then freeze-dried to obtain crude polysaccharide product. The crude product was dissolved in distilled water and trichloroacetic acid (TCA) (10% w/v) was added to remove its protein impurities by centrifugation (6000 rpm for 10 min) and 2 or 3 repeats of the reaction. The supernatant was collected was and dialyzed against distilled water for 24 h and then freeze-dried to obtain the pure polysaccharide of *M. veneriformis* (MVPS). The MVPS is an opalescent powder and was measured to have an over 97.3% total sugar content by reference to D-glucose *via* the anthrone-sulfuric acid method (Laurentin and Edwards, 2003; Piccolo et al., 2008).

The product Ah-MVPS were correspondingly prepared by incomplete degradation of HCl, as reported in our previous study (Wang et al., 2016). In brief, about 2.0g MVPS was dissolved in 100 mL distilled water to prepare 20 mg/mL of solution. Ten milliliter MVPS solutions were carefully transferred into a tapered flask (50 mL) and 1.0 mol/L of HCl was added under ice-bath conditions. Afterward, the flasks were transferred to a water bath (80 °C) to initiate the acidic hydrolyzing reaction, without agitation. The reaction lasted for 2 h and was then stopped by neutralization with NaOH (10 mol/L) containing a small amount of sodium borohydride (0.05 mol/L). The hydrolysates were supplemented with a two-fold volume of ethanol and centrifuged at 12000 rpm for 10 min to remove the generated salts or impurities. One final products, termed Ah-MVPS, were retrieved by freeze-drying and were preserved in a glass desiccator for further use.

### Analysis of Ah-MVPS

About 100 mg of Ah-MVPS were precisely weighted and dissolved in 100 mL distilled water to prepare 1.0 mg/mL of stock solution. Ah-MVPS stock solution was diluted to anticipative concentrations to determine the content of total sugar and reducing sugar. The total sugar content was measured by the anthrone-sulfuric acid method (Laurentin and Edwards, 2003; Piccolo et al., 2008), while the reducing sugar content was quantified by the 3,5-dinitrosalicylic acid (DNS) method in calibration with D-glucose (Zhao et al., 2008). The oligosaccharides of Ah-MVPS were initially analyzed by high-performance liquid chromatography (HPLC), adopting the methods of Chen (Chen et al., 2004) and using a Waters ™ Alliance 2695 HPLC Separation Module in combination with a Shodex Asahipak NH2P-4E column (4.6mm×250mm) and a Dionex PA-1 guard column (4.0mm×50mm). Further identification of the oligosaccharides of Ah-MVPS was executed on an HPLC-ESI-TOF-MS system. The system consisted of two successive coupled apparatuses: a Waters 2695 HPLC Separation Module equipped with a Shodex Asahipak NH2P-4E column (4.6mm×250mm) and a Time-of-Flight Mass Spectrometer (QTOF-MS, Waters QTOF Premier) equipped with an electrospray source.

### Animal experiments

#### Animals and Grouping

The Kunming strain mice (male, 8-week-old, 20 ± 2 g), purchased from the Experimental Animal Research Center of Nanjing Medical University (Certificate no. SCXKC (Su) 2008-2014), were housed in cages and kept under conditions of a 12 h light/dark cycle, 22 ± 2°C, 50-55% humidity, and free access to water and standard food. All experiments were performed following the Regulations of Experimental Animal Administration issued by the State Committee of Science and Technology of the People’s Republic of China.

After a week of acclimatization, all mice were randomly divided into six groups (eight mice per group), including one normal control (NC) group, one model control (MC) group, one positive control (PC) group, and three treatment groups. The mouse grouping reflected the different gavage procedures. During the animal experiment, mice in the three treatment groups received Ah-MVPS once daily at doses of 62.5, 125, and 250 mg/kg-b.w., mice in the PC group were administrated with a well-known liver-protective reagent, bifendatatum (150 mg/kg-b.w.), that is commonly used to treat the transaminase elevation caused by viral hepatitis, while mice in the NC and MC groups only received an equivalent volume of saline.

#### Acute Hepatic Damage Induced by CCl_4_

For this experiment, the groups of Kunming mice were processed by intragastric gavage (i.g.) once daily and continued for 7 successive days. Eight hours after the last gavage, all animals except for the NC group were intraperitoneally administered (10 mL/kg-b.w.) a CCl4/peanut oil mixture (0.1% v/v) to induce acute hepatic injury, while the NC group mice only received the same volume of physiological saline at that time (**Figure 2A**). All mice were then fasted but allowed to drink water as usual.

#### Acute Hepatic Damage Induced by Ethanol

In another experiment, all mice received test samples once daily for 25 successive days *via* intragastric gavage according to a protocol. From day 26 for the next consecutive 7 days, all mice except the NC group were orally intoxicated (15 mL/kg-b.w.) with intense liquor (Hongxing Erguotou^®^, 56%v/v of alcohol) after 8 h of the normal treatment once daily, while the NC group received an equal volume of physiological saline instead of liquor (**Figure 3A**). During this experiment, all animals were fasted but permitted to drink water as usual.

#### Detections of Biological Indicators of Hepatic Damages

After 4 h of the CCl_4_ intoxication or 8 h of the last liquor intoxication of animals in the above two experiments, about 0.5 mL of blood was collected from each mouse by excising the eyeballs to acquire the serum (seen in **Figures 2A** and **3A**). ALT and AST levels in the serum were measured using standard enzymatic assays according to the kit protocols (Nanjing Jiancheng Bioengineering Institute, China). All mice were subjected to cervical detachments at another eight hours after the blood collection. At that time point, the peritoneal cavity was opened along the abdominal middle line, and the liver was carefully isolated and removed to calculate the liver index (wet liver weight/body weight×100%). Partial hepatic tissues were then cut off from the whole liver specimen and homogenized (1:9, w/v) in saline after being washed with cold saline. The SOD activities and contents of MDA, calibrating to the protein measurement of the hepatic tissue, were analyzed using a commercial kit according to the instructions.

#### Histological Examination

Mouse liver specimens were immersed in PBS buffer containing 10% formalin (pH 7.4) for 24 h and embedded in paraffin. Thin sections were prepared by using a microtome and stained with hematoxylin-eosin (H&E). Each section was photographed under a microscope to show the histopathological changes in the liver. Images of each tissue slice were taken at a magnification of 400×. At the same time, pathological scoring was performed for the liver tissues to access their degree of injury with the help of two experts. The liver specimens were classed into five degrees of injury according to their tissue lesions under optical observation, including slight (< 5%), mild (5~15%), moderate (15~30%), significant (30~50%), and severe (≥50%) lesions. Six out of eight mice in each group were evaluated with the pathological scoring.

### Cell Experiments

#### Cell Culturing

L-02 human normal liver cells were obtained from the cell bank of the pharmacology and toxicology room of Nanjing University of Traditional Chinese Medicine. The normal human hepatocyte cell line L-02 was cultured in DMEM medium supplemented with 10% FBS, 100 IU/mL of penicillin, and 100 μg/mL of streptomycin. Cells were incubated in a humidified atmosphere of 5% CO_2_ at 37°C and passaged according to the recommended procedures of the ATCC (American Type Culture Collection). They were used for experiments from the logarithmic phase of growth, seeded into 96-well plates (5×10^3^ cells per well, 180 μL).

#### Cell Viability Assay

L-02 cells were seeded into a 96-well plate at a density of 5000 cells per well and incubated for 24 h. Varying concentrations of Ah-MVPS pre-dissolved in culture media were then added to the L-02 cells. Cell proliferation after another 4 h of co-incubation was assayed using the MTS/PMS method. Briefly, 100 μL of MTS/PMS working solution was added to each well and then incubated at 37°C for 1 h. The absorbance of each well was measured at 490 nm using a full-length microplate reader (Beckman AD-340, USA).

#### Determinations of Protection Against Cytotoxicity

We here examine the protective effects of Ah-MVPS on H_2_O_2_- or ethanol-induced L-02 cell damage. In these examinations, L-02 cells were seeded on 96-well plates (2.0×10^4^ per well) and randomly divided into eight sets (ten wells per set) including normal control (NC), model control (MC), and positive control (PC) wells, as well as five sets with various concentrations of Ah-MVPS. The cultured cells were then supplemented with 200 μL of different reagents for pre-incubation. The PC wells had 50 μg/mL bifendatatum in culture media added to them, and the wells for As-MVPS sets had 20, 40, 80, 160, or 320 μg/mL of the tested sample added to them, while the NC and MC wells received only blank culture media of equivalent volume. After 2 h of incubation (37°C, 5% CO_2_), 200 μL of H_2_O_2_ (10% v/v) or ethanol (50% v/v) as the cytotoxic agent was add into each well of all sets except NC, for which the toxicants were replaced with an equivalent volume of culture medium (Junming et al., 2015). The L-02 cells were grown for another 8 h, and finally, each well was investigated for corresponding biochemical markers that can reflect cell damages. Supernatants (0.2 mL) above the cultured cells were withdrawn, and the activities of ALT and AST in them were determined using the commercial enzymatic assay kits according to standard protocols. The cells were also collected after removing the supernatants, and Triton-100 (0.2 mM) was then used to lyse the cells and release cytoplasm. After that, MDA content and SOD activity were determined by the standard commercial kit by using standard protocols and calibrated with the protein measurement of L-02 cells, respectively.

### Statistical Analysis

All the data are expressed as the mean ± S.D. The data were assessed using professional statistics software (SPSS version 19.0, Chicago, IL, USA). A non-parametrial Kolmogorov-Smirnov test was employed for verifying a normal distribution of the data. Differences between groups were assessed by one-way analysis of variance (ANOVA) with either LSD (assuming equal variances) or Dunnett’s T3 (not assuming equal variances) for posthoc analyses. Statistical significance was recognized at p < 0.05.

## Results

### Chemical Characterization of Ah-MVPS

**Figure 1:**
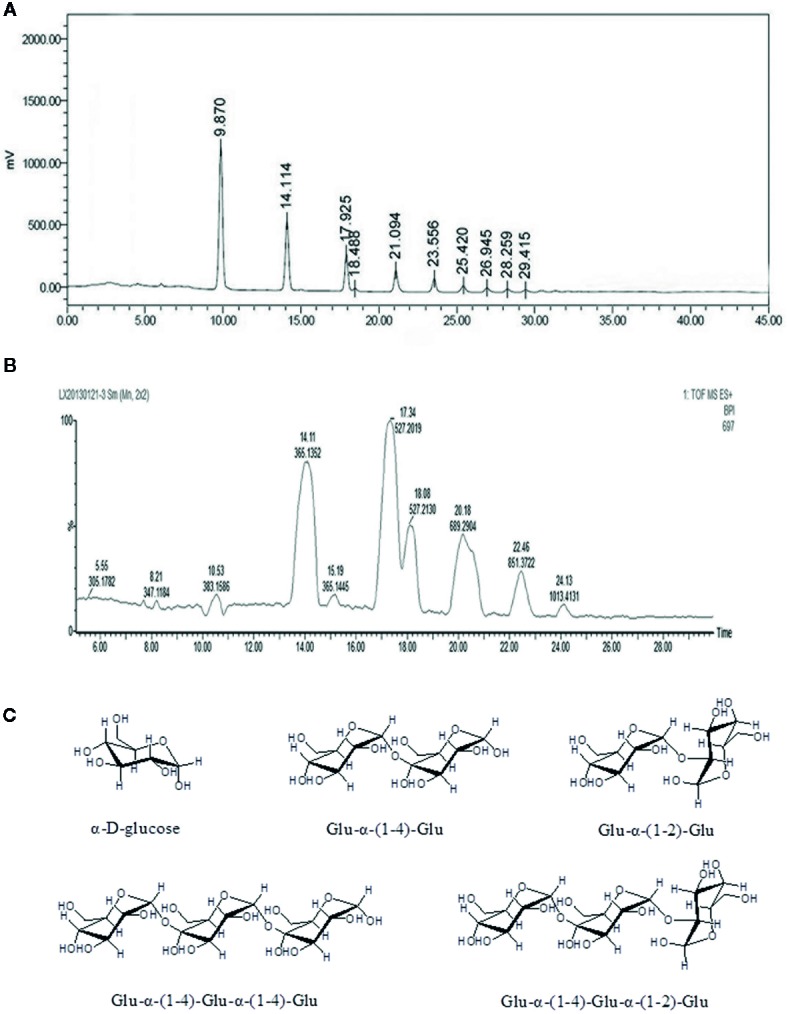
Chemical composition analysis of the Ah-MVPS exhibited by HPLC profile **(A)** and the total ion flow chromatography of LC-QTOF-MS **(B)**, and structural drawing of representative oligosaccharides detected in the Ah-MVPS product **(C)**.

The Ah-MVPS product mainly consisted of numerous oligosaccharide molecules with various degrees of polymerization (DP) because it was derived from MVPS polysaccharide. Almost all ingredients in the Ah-MVPS product are short-chain sugars. Its total sugar content is near to 98.7%, and the reducing sugar content was determined to be 32.8%. Its oligosaccharide compositions were examined with sugar-affinity column (NH2P-4E) coupled HPLC, and nine oligomer ingredients were detected in the sample (**Figure 1A**). Its oligosaccharides were subsequently identified with the LC-QTOF-MS analysis (**Figure 1B**), and seven oligomers were recognized from their structural attributions in chemistry due to retrieval of their MS signal responses. This mass identification showed the Ah-MVPS to be mainly composed of α-D-glucose and Glu-α-(1-4)-Glu and/or Glu-α-(1-2)-Glu dimers, as well as Glu-α-(1-4)-Glu-α-(1-4)-Glu or Glu-α-(1 -4)-Glu-α-(1-2)-Glu as trimers. Their chemical structures were completely drafted and are shown in **Figure 1C**. There are 42.6% of Glu, 27.5% of dimers, and about 18.7% of trimers in the molar composition of Ah-MVPS, as calculated from the peak areas of the HPLC curve. Other oligosaccharides with higher DP might be minor components of Ah-MVPS and are no reported on here because they made up only 11.1% of the composition.

### Effects of Ah-MVPS on Acute CCl_4_ Induced Liver Injury

**Figure 2:**
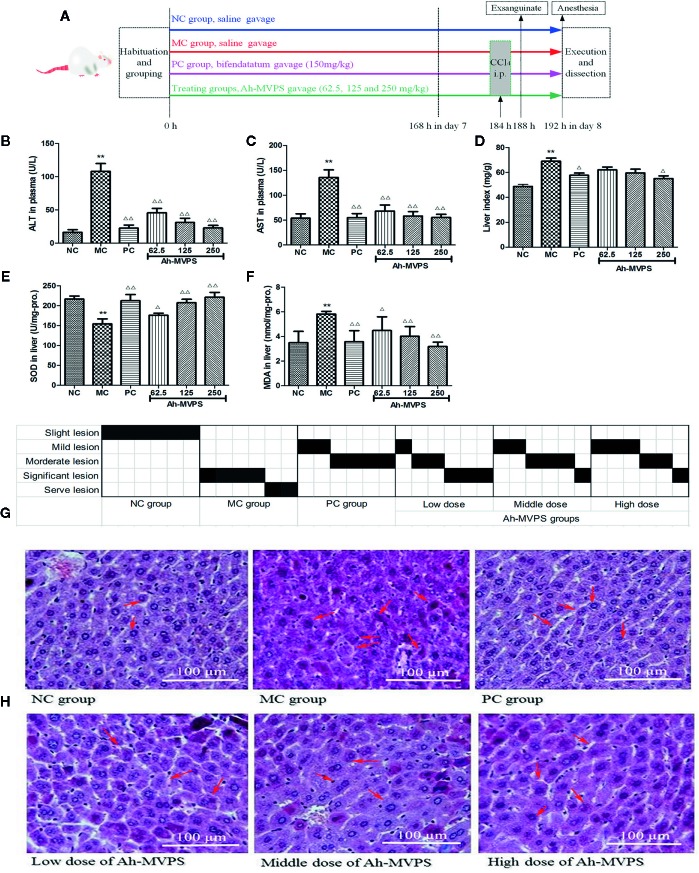
Protective effects of Ah-MVPS on the injury of mouse liver induced by CCl_4_. The time protocols of animal disease model setting and administration **(A)**. Comparative analysis of ALT (serum alanine aminotransferase, **B**), AST (aspartate transaminase, **C**), LI (liver index, **D**), SOD (superoxide dismutase, **E**), and MDA (malondialdehyde, **F**) of mice in each group, **P < 0.01 vs. normal control (NC) group, ^Δ^P < 0.05, ^ΔΔ^P < 0.01 vs. model control (MC) group. Recording table of pathological scoring **(G)**, and typical H&E section images **(H)** of mouse liver tissues (400-fold magnification); red arrows indicate normal and/or injured hepatocytes.

Oral administration of Ah-MVPS could effectively attenuate the increase of serum alanine aminotransferase (ALT) and aspartate transaminase (AST) vitality that resulted from liver injury due to the injection of CCl_4_. As depicted in **Figures 2B, C**, the enzymatic activities of serum AST and ALT of model control (MC) mice dramatically increased to 136 and 110 U/L (p < 0.01) from 54 and 14 U/L in the normal control (NC) mice, correspondingly. When the dose of Ah-MVPS was increased to 125 mg/kg-b.w., AST and ALT vitality dropped to 58 and 30 U/L (p < 0.01, *vs.* MC mice), and at a further elevating dose with 250 mg/kg-b.w. of Ah-MVPS, serum AST and ALT activities of mice were depressed to 55 and 23 U/L, individually (p < 0.01). In this experiment, bifendatatum as positive control also sharply reduced the enzyme activities of serum AST and ALT by 59.5% and 83.9% relative to CCl_4_-intoxicated mice (p < 0.01). As shown in **Figure 2D**, Ah-MVPS reduced the liver index in a dose-dependent manner.

The measurements for hepatic superoxidase dismutase (SOD) activity in all mice are displayed in **Figure 2E**. A single application of CCl_4_ led to a significant reduction of SOD, with the SOD levels in liver of MC mice decreased to 155 U/mg-protein (p < 0.01) from 219 U/mg-protein in liver of NC mice (without injection of CCl_4_). The reduction of hepatic SOD activity in CCl_4_-intoxicated groups was weakened by pretreatment with Ah-MVPS at 125 and 250 mg/kg-b.w. (p < 0.01), and the SOD-promoting effect of Ah-MVPS exhibited dose-dependent behavior. Furthermore, the levels of SOD activity-promotion caused by middle or high dosage of Ah-MVPS gavages are basically the same to that of bifendatatum as positive SOD accelerant at doses of 150 mg/kg, the effect of which is obvious in inhibiting the reduction of SOD activity in liver tissue with CCl_4_-induced hepatic injuries (p < 0.01 *vs.* MC mice). The generation of malondialdehyde (MDA) can be used to signify the lipid peroxidation levels in liver (Maheshwari et al., 2011); thus, detection of the MDA content of liver tissue was focused upon in our research. The measured results for mouse hepatic MDA are shown in **Figure 2F**. The hepatic MDA levels of the NC mice were 3.5nmol/mg-protein. However, the liver MDA levels of CCl_4_-induced mice (MC) were remarkably elevated to 5.8nmol/mg-protein (p < 0.01 *vs.* NC). Pretreatment with Ah-MVPS at doses of 62.5, 125, and 250 mg/kg-b.w. to mice could effectively decrease the CCl_4_-induced production of MDA (p <0.01 *vs.* MC); this accords with prior expectations. Moreover, an obvious dose-dependent MDA inhibition was observed in the Ah-MVPS groups, and a higher dose had a more significant effect, near that of bifendatatum at a dose of 250 mg/kg. The bifendatatum was used as a positive hepatoprotective drug and could also prominently inhibit the elevation of MDA to 3.6nmol/mg-protein. These results suggest that Ah-MVPS may prevent liver against chemical damage by suppressing lipid peroxidation *in vivo*.

Liver protection by Ah-MVPS could also be confirmed through histopathological examinations of the morphological changes of liver tissue derived from CCl_4_-injured mice. As showed in **Figure 2H**, H&E tissue slices of liver of mice in the NC group exhibited a normal cellular architecture with clear hepatic cells, a central vein, and sine spaces. In contrast, quite serious damage can be seen in a slice from the MC group, whose H&E image shows severe hepatocyte necrosis, massive fatty changes, hepatocellular swelling, water-like deterioration of hepatocytes, loss of cellular boundaries, and collapse of reticular scaffolds, as well as the formation of eosinophils and broad infiltration of lymphocytes. However, liver tissue belonging to Ah-MVPS-treated mice showed a repaired liver lobular H&E morphology by exhibiting a slight hepatic fatty change, less necrosis, and lobular inflammation as compared with the liver H&E slices of the NC group. These results demonstrate that Ah-MVPS can protect liver tissue from acute CCl_4_-intoxication hepatic damage (**Figure 2H**). A more intuitive result is displayed in **Figure 2G**, which gives semi-quantitative values (pathological score) to describe the degrees of damage to mice livers of the six groups. Six mice per group were examined, and the value of each was marked in black on the scoring sheet. It is exhibited that the degree of liver damage in the mice was in the order of MC > low dose of Ah-MVPS > middle dose of Ah-MVPS > high dose of Ah-MVPS > PC > NC. The dose-dependent liver protective effect of Ah-MVPS is clearly obvious in this ranking.

### Effects of Ah-MVPS on Acute Alcoholic Liver Injury

**Figure 3:**
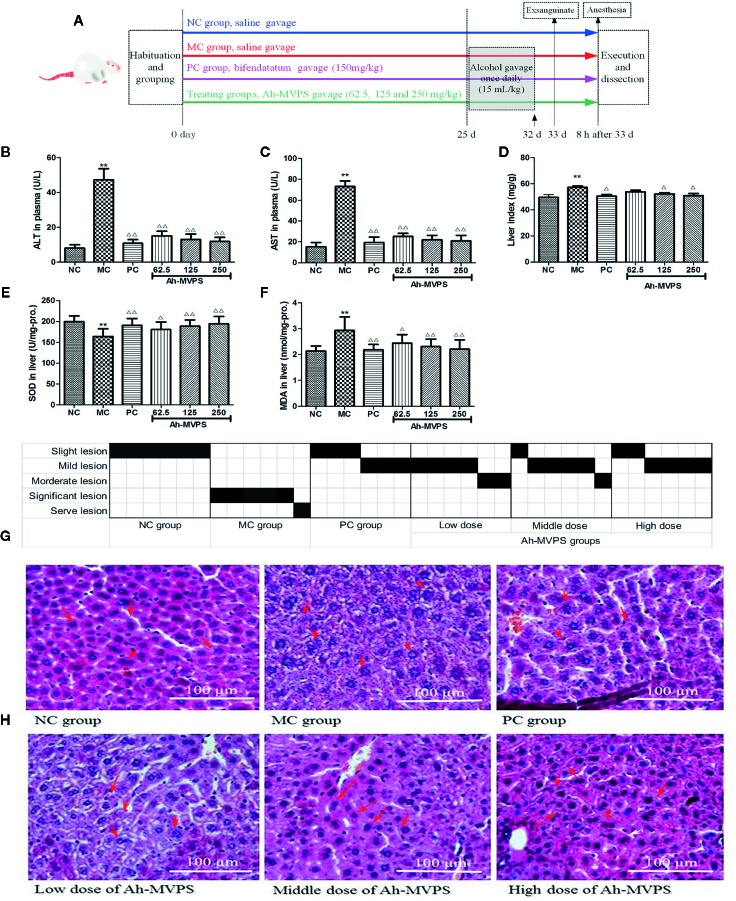
Protective effects of Ah-MVPS on the injury of mouse liver induced by alcohol. The time protocols of animal disease model setting and administration **(A)**. Comparative analysis of ALT (serum alanine aminotransferase, **B**), AST (aspartate transaminase, **C**), LI (liver index, **D**), SOD (superoxide dismutase,** E**), and MDA (malondialdehyde, **F**) of mice in each group, **P < 0.01 vs. normal control (NC) group, ^Δ/^P < 0.05, ^ΔΔ^P < 0.01 vs. model control (MC) group. Recording table of pathological scoring **(G)**, and typical H&E section images **(H)** of the mouse liver tissues (400-fold magnification); red arrows indicate normal and/or injured hepatocytes.

After the relatively long term gavage, mice exhibited unusual resistance to alcoholic damages, preserving their liver morphology and function. This animal experiment provided further information besides showing the definite effectiveness of Ah-MVPS. Firstly, an alcoholic-liver-injured animal model was established successfully. It was seen that the serum ALT and AST levels (**Figures 3B, C**), liver index (**Figure 3D**), and hepatic MDA content (**Figure 3E**) and SOD activity (**Figure 3E**) of MC mice were obviously increased as compared to NC mice (P < 0.01), which was also reflected by the relatively serious lesions found in mouse liver under histological examination (**Figure 3G**). The pathological consequences were that the hepatic cords in the liver tissues of MC mice exhibited irregular lobules in combination with membrane destruction of hepatocyte boundaries, collapse of reticular scaffolds, and cytoplasmic leakages (**Figure 3H**). Secondly, the liver protection effect of Ah-MVPS pre-gavage continued for 25 days was confirmed through a comparison of biochemical and histological indexes between the subjects and MC mice. Serum ALT and AST of every Ah-MVPS group were significantly decreased by 2~4 folds compared to MC mice (P < 0.01), and they reached the level of the PC group. The significant drop in serum transaminase reflects the ability of Ah-MVPS to moderate the hepatic damages caused by excessive alcohol intake and protect liver functions in a dose-dependent manner. Administration of Ah-MVPS to mice not only reduced the liver index (**Figure 3D**) and MDA content (**Figure 3F**) but also increased the SOD activity (**Figure 3E**) of mouse livers. The liver index and hepatic MDA levels declined markedly in Ah-MVPS-treated and PC mice as compared to the MC. Moreover, liver SOD activity was enhanced in Ah-MVPS-treated groups and PC groups. The reduction in MDA content and liver index, as well as the increase in SOD activity, are extremely significant in the middle and high Ah-MVPS dose groups (P < 0.01), and the high-dose treatment even resulted in a stronger effect than in the positive control (PC) group. In histological observation, the liver cells of mice administered a high dose of Ah-MVPS were arranged regularly, and the ballooning degeneration of liver cell cytoplasm was alleviated (**Figure 3H**). Finally, Ah-MVPS are more suitable for protecting liver from alcoholic damage than against CCl_4_ intoxication based on comparison of the direct data (**Figures 2** and **3**), which might correlate with the low level of injuries in intoxicated liver and long-term of administration with Ah-MVPS. It was noted from the pathological scoring that two mice were restored to no tissue lesions, while the other four examined mice had only mild liver lesions after receiving 250 mg/kg of Ah-MVPS (**Figure 3G**). This beneficial result was hard to obtain for the CCl_4_-induced serve liver injury in mice, indicating that Ah-MPVS might be a suitable antagonist of ethanol.

### Effects of Ah-MVPS on the Injury of L-02 Cells Induced by H_2_O_2_ or Ethanol

**Figure 4:**
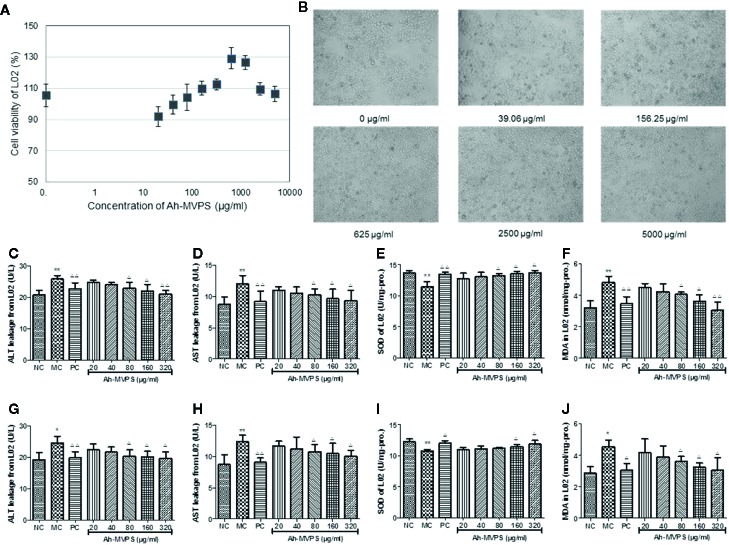
Protection of Ah-MVPS against chemical damage to L-02 cell. The influence of MVPS concentrations on L-02 cell viability and after 24h of co-incubation **(A)**, and optical images of L-02 incubated with MVPS **(B)**. Quantitation of leakage of ALT (serum alanine aminotransferase, **C, G**) and AST (aspartate transaminase, **D, H**), SOD (superoxidase dismutase, **E, I**) activity, and MDA (malondialdehyde, **F, J**) content for L-02 cells injured by H_2_O_2_ and ethanol, respectively; *P< 0.05, **P < 0.01 vs. normal control (NC) set, ^Δ^P < 0.05, ^ΔΔ^P < 0.01 vs. model control (MC) set.

There was no obvious influence of the addition of Ah-MVPS on the viability of L-02 cells (**Figure 4A**). Under co-incubation with Ah-MVPS, L-02 cells fluctuated within 90~130% of viability under lab setting conditions, indicating that the Ah-MVPS are not toxic to hepatocytes over a wide range of concentrations in cell assays. This conclusion can be corroborated by the optical observations (**Figure 4B**), in which the density and morphology of L-02 cells were hardly altered by supplementation of Ah-MVPS in the concentrations range 0~5000 μg/mL.

However, supplementation of Ah-MVPS in concentrations of 80~320 μg/mL can effectively prevent damage to L-02 cells aroused by H_2_O_2_ (**Figures 4C–F**) or ethanol (**Figures 4G–J**). These results indicate that Ah-MVPS could decrease the cell damage by the suppression of ALT and AST leakage, promotion of SOD activity, and inhibition of MDA production. The protective effects were increased at increased concentrations of Ah-MVPS. At a concentration of 320μg/mL, the effect of Ah-MVPS is equivalent to that of bifendatatum, the positive control.

## Discussion

In our previous research (Wang et al., 2016), we degraded MVPS by mild H_2_SO_4_ or HCl hydrolysis and found that hydrolysates were extremely influenced by adding an amount of acid and by the reaction time. To avoid thorough hydrolysis and obtain more oligosaccharide content in the product, degradation with 0.5 h of HCl was adopted to process MVPS due to its controllability. HCl hydrolysates contained many oligosaccharides that could be considered active ingredients. The superior ability of Ah-MVPS in scavenging hydroxyl radicals was confirmed by activity screening among polysaccharides and various hydrolysates. We also elucidated the composition and content of oligosaccharides in the Ah-MVPS, which include many oligomers of glucan that are varied in DP and linkages, as shown in **Figure 1**. The results were especially noteworthy in that linkages of α-(1-2) residues were identified in oligomers of Ah-MVPS, which would be exploitable for drug candidates functioning in a new way. However, there is still a long way to go to clarify its functions and mechanism due to a lack of data accumulation.

In this study, we exhibit the liver-protective activities of the HCl-hydrolysis products of marine clam polysaccharide (Ah-MVPS). A suitable animal model is a good basis for investigation of the pathogenesis and pathology of liver injury and is also an effective way of screening active ingredients. The toxic reagents CCl_4_ and ethanol are commonly used at present to establish acute chemical liver injury in mice, though the potential model designs are diverse in terms of animal strain, administration route, dose, and time of exposure (Liu et al., 2015; Zhou et al., 2017). For our experiment, the administration route, time of exposure, and dosage of the model established were decided by literature review, pre-experiments, and screens. One acute alcoholic liver injury model was established by the oral gavage route to cause mouse liver function loss so significant that it accords with human drinking habits, which avoided the shortcomings usually arising with intraperitoneal injections, such as organ adhesion, infection, and ascite formation (Wang et al., 2012; Zhao et al., 2017). However, the other acute liver injury model was induced by intraperitoneal CCl_4_ injection of mice since the toxicant was absorbed quickly, and just one injection would bring an overall toxic response to animals. Otherwise, many repeats of oral administration CCl_4_ are required by a combination of peanut oil as a solvent, which usually results in diarrhea. Diarrhea not only pollutes the environment but can also easily cause secondary absorption through the mouth and the skin, generating a large dose error (Zeng et al., 2017; Sun et al., 2018a). Oxidative stress is commonly recognized as indispensable factor for exploring injury mechanisms in most chemical liver injury models (Recknagel and Glende, 1989; Sun et al., 2018a; Sun et al., 2018b). Therefore, we chose a very representative oxidative stress injury model induced by H_2_O_2_ and/or ethanol to further evaluate the protective activities of Ah-MVPS in cellular experiments in association with comparing with the results of bifendatatum as the positive drug. It has an obvious antioxidant effect, inhibiting hepatic lipid peroxidation by stabilizing the hepatocyte membrane, alleviating the structural damage of hepatic tissue, and reducing the ALT and AST leakages arising due to various pathogenic damages, such as CCl_4_ and ethanol intoxication of hepatocytes (Liu et al., 2016; Jing et al., 2017; Song et al., 2018).

It was finally confirmed in this study that Ah-MVPS have excellent liver-protective activities that perhaps correlate with their antioxidant nature as oligosaccharides that are complex in chemistry. To some extent, the liver protection effect of Ah-MVPS is even stronger than that of other similar products that are already well known. For example, enzymatic hydrolysates of the polysaccharide extract (EPS) from *Pleurotus eryngii* exhibited significant hepatoprotective activities to CCl_4_-injured mice (Zhang et al., 2017) only when its dose was near to 400 mg/kg-b.w. and it was administrated by continuous oral gavage for 28 days. In another study, enzymatic and acidic hydrolysates of polysaccharides isolated from *Pleurotus geesteranus* mycelium were also effective in protecting mouse liver against injury by ethanol, but the efficiency was not obvious unless the dose was increased to 200 mg/kg-b.w. and treatment course was extended to 25 days (Song et al., 2018). In our study, just seven days of Ah-MVPS treatment to mice by oral gavage at a dose of 62.5 mg/kg-b.w. can significantly protect the liver against CCl_4_-induced injuries. Hepatoprotection of Ah-MVPS at the high dose (250 mg/kg) is essentially equal to that of bifendatatum (250 mg/kg) in the mouse model. The liver protective effect of Ah-MVPS was subsequently authenticated again in ethanol-induced liver-damaged mice and chemically injured hepatic parenchymal cells. Further investigations may focus on the specific oligosaccharide ingredients in the Ah-MVPS product, such as Glu-α-(1-2)-Glu or Glu-α-(1-4)-Glu-α-(1-2)-Glu, whose liver-protective effects could be confirmed by rapid comparison and screening in animals or cellular experiments. The biochemical mechanism also needs to be considered as a part of the comprehensive research so as to interpret the roles of the specific oligosaccharides in liver protection.

## Conclusion

In conclusion, our results confirm the preventative effects of Ah-MVPS against hepatic injury both *in vivo* and *in vitro*. The defensive mechanism of Ah-MVPS is, at least in part, related to the improvement of oxidative stress and lipid peroxidation. Taken together, our results show that Ah-MVPS have the potential to be developed as pharmaceutical or functional ingredients to benefit patients suffering from liver injury.

## Data Availability Statement

The datasets generated for this study are available on request to the corresponding author.

## Ethics Statement

The animal study was reviewed and approved by Laboratory Animal Welfare Ethics Committee in Nanjing University of Chinese Medicine.

## Author Contributions

LW and YF conceived and designed the project. LW and YY performed experiments and wrote the manuscripts. H-YT and SL analyzed the data and gave some helpful advice.

## Funding

This work has been sponsored financially by the Major Project of Natural Science Research in Universities of Jiangsu Province (No. 18KJA360008) and Jiangsu Overseas Visiting Scholar Program for University Prominent Young & Middle-aged Teachers and Presidents in 2018 (Approved No. [2018]4156). This work was also co-supported by the Research Grants Committee of Hong Kong (project codes: 740608, 766211 and 17152116) and the Innovation Technology Fund of Hong Kong (ITF. Project code: 260900263). We would also like to thank Dr. Ning Wang (School of Chinese medicine, the University of Hong Kong) for the helpful discussion.

## Conflict of Interest

The authors declare that the research was conducted in the absence of any commercial or financial relationships that could be construed as a potential conflict of interest.

## References

[B1] Chen H. M. Zheng L. Lin W. Yan X. J. (2004). Product monitoring and quantitation of oligosaccharides composition in agar hydrolysates by precolumn labeling HPLC. Talanta 64, 773–777. 10.1016/j.talanta.2004.04.002 18969671

[B2] Gu X. Manautou J. E. (2012). Molecular mechanisms underlying chemical liver injury. Exp. Revi. Mol. Med. 14, e4. 10.1017/s1462399411002110 PMC3704158 22306029

[B3] Jing L. Y. Zone S. Li J. L. Ye M. Surahio M. Yang L. (2017). Potential mechanism of protection effect of exopolysaccharide from Lachnum YM406 and its derivatives on carbon tetrachloride-induced acute liver injury in mice. J. Funct. Foods 36, 203–214. 10.1016/j.jff.2017.06.057

[B4] Junming W. Yueyue Z. Ruixin L. Xiaobing L. Ying C. Lingbo Q. (2015). Geniposide protects against acute alcohol-induced liver injury in mice via up-regulating the expression of the main antioxidant enzymes. Can. J. Physiol. Pharmacol. 93, 261–267. 10.1139/cjpp-2014-0536 25730420

[B5] Laurentin A. Edwards C. A. (2003). A microtiter modification of the anthrone-sulfuric acid colorimetric assay for glucose-based carbohydrates. Anal. Biochem. 315, 143–145. 10.1016/S0003-2697(02)00704-2 12672425

[B6] Liu Y. J. Du J. L. Cao L. P. Jia R. Shen Y. J. Zhao C. Y. (2015). Anti-inflammatory and hepatoprotective effects of Ganoderma lucidum polysaccharides on carbon tetrachloride-induced hepatocyte damage in common carp (Cyprinus carpio L.). Int. Immunopharmacol. 25, 112–120. 10.1016/j.intimp.2015.01.023 25639226

[B7] Liu M. Meng G. Y. Zhang J. J. Zhao H. J. Jia L. (2016). Antioxidant and Hepatoprotective Activities of Mycelia Selenium Polysaccharide by Hypsizigus marmoreus SK-02. Biol. Trace Element Res. 172, 437–448. 10.1007/s12011-015-0613-z 26743865

[B8] Liu S. Wang Q. K. Song Y. F. He Y. H. Ren D. D. Cong H. H. (2018). Studies on the hepatoprotective effect of fucoidans from brown algae Kjellmaniella crassifolia. Carbohydr. Polym. 193, 298–306. 10.1016/j.carbpol.2018.03.077 29773385

[B9] Luan H. M. Wang L. C. Wu H. Jin Y. Ji J. (2011). Antioxidant activities and antioxidative components in the surf clam, Mactra veneriformis. Nat. Product Lett. 25, 1838–1848. 10.1080/14786419.2010.530268 21756123

[B10] Maheshwari D. T. Yogendra Kumar,. M. S. Verma S. K. Singh V. K. Som Nath S. (2011). Antioxidant and hepatoprotective activities of phenolic rich fraction of Seabuckthorn (Hippophae rhamnoides L.) leaves. Food Chem. Toxicol. 49, 2422–2428. 10.1016/j.fct.2011.06.061 21723907

[B11] Muriel P. (2009). Role of free radicals in liver diseases. Hepat. Int. 3, 526–536. 10.1007/s12072-009-9158-6 PMC2790593 19941170

[B12] Piccolo A. Zena A. Conte P. (2008). A comparison of acid hydrolyses for the determination of carbohydrate content in soils. Commun.Soil Sci. Plant Anal. 27, 2909–2915. 10.1080/00103629609369749

[B13] Poli G. (1993). Liver damage due to free radicals. Br. Med. Bull. 49, 604–620. 10.1093/oxfordjournals.bmb.a072634 8221026

[B14] Pushpavalli G. Kalaiarasi P. Veeramani C. Pugalendi K. V. (2010). Effect of chrysin on hepatoprotective and antioxidant status in -galactosamine-induced hepatitis in rats. Eur. J. Pharmacol. 631, 36–41. 10.1016/j.ejphar.2009.12.031 20056116

[B15] Recknagel R. O. Glende,. E. G. Jr. (1989). Mechanisms of carbon tetrachloride toxicity. Pharmacol. Ther. 43, 139–154. 10.1016/0163-7258(89)90050-8 2675128

[B16] Song X. L. Liu Z. H. Zhang J. J. Zhang C. Dong Y. H. Ren Z. Z. (2018). Antioxidative and hepatoprotective effects of enzymatic and acidic-hydrolysis of Pleurotus geesteranus mycelium polysaccharides on alcoholic liver diseases. Carbohydr. Polym. 201, 75–86. 10.1016/j.carbpol.2018.08.058 30241865

[B17] Sun Y. F. Yang X. B. Lu X. S. Wang D. Y. Zhao Y. (2013). Protective effects of Keemun black tea polysaccharides on acute carbon tetrachloride-caused oxidative hepatotoxicity in mice. Food Chem. Toxicol. 58, 184–192. 10.1016/j.fct.2013.04.034 23623843

[B18] Sun J. Wen X. Y. Liu J. Kan J. Qian C. L. Wu C. S. (2018a). Protective effect of an arabinogalactan from black soybean against carbon tetrachloride-induced acute liver injury in mice. Int. J. Biol. Macromol. 117, 659–664. 10.1016/j.ijbiomac.2018.05.203 29852225

[B19] Sun J. Zhou B. Tang C. Gou Y. R. Chen H. Wang Y. (2018b). Characterization, antioxidant activity and hepatoprotective effect of purple sweetpotato polysaccharides. Int. J. Biol. Macromol. 115, 69–76. 10.1016/j.ijbiomac.2018.04.033 29653172

[B20] Tong C. Q. Zheng Y. X. Guo G. L. Li W. (2015). Hepatoprotective effect of a polysaccharide from Auricularia auricula root on acute model of liver injury. Glycobiology 25, 1287–1287.

[B21] Uyanoglu M. Canbek M. Van Griensven L. J. L. D. Yamac M. Senturk H. Kartkaya K. (2014). Effects of polysaccharide from fruiting bodies of Agaricus bisporus, Agaricus brasiliensis, and Phellinus linteus on alcoholic liver injury. Int. J. Food Scie. Nutr. 65, 482–488. 10.3109/09637486.2013.869796 24392995

[B22] Wang L. Wu H. Chang N. Zhang K. (2011a). Anti-hyperglycemic effect of the polysaccharide fraction isolated from mactra veneriformis. Front. Chem. Sci. Eng. 5, 238–244. 10.1007/s11705-010-0002-2

[B23] Wang L. C. Zhang K. Di L. Q. Liu R. Wu H. (2011b). Isolation and structural elucidation of novel homogenous polysaccharide from Mactra veneriformis. Carbohydr. Polyme. 86, 982–987. 10.1016/j.carbpol.2011.05.052

[B24] Wang M. C. Zhu P. L. Jiang C. X. Ma L. P. Zhang Z. J. Zeng X. X. (2012). Preliminary characterization, antioxidant activity in vitro and hepatoprotective effect on acute alcohol-induced liver injury in mice of polysaccharides from the peduncles of Hovenia dulcis. Food Chem. Toxicol. 50, 2964–2970. 10.1016/j.fct.2012.06.034 22750723

[B25] Wang L. Wang X. Wu H. Liu R. (2014). Overview on Biological Activities and Molecular Characteristics of Sulfated Polysaccharides from Marine Green Algae in Recent Years. Marine Drugs 12, 4984–5020. 10.3390/md12094984 25257786 PMC4178480

[B26] Wang L. C. Wu H. Ji J. Xue F. Liu R. (2016). Preparation, analysis and antioxidant evaluation of the controlled product of polysaccharide from Mactra veneriformis by mild acid hydrolysis. Carbohydr. Polym. 137, 709–718. 10.1016/j.carbpol.2015.11.030 26686183

[B27] Wang L. C. Di L. Q. Li J. S. Hu L. H. Cheng J. M. Wu H. (2019). Elaboration in type, primary structure, and bioactivity of polysaccharides derived from mollusks. Crit. Rev. Food Sci. Nutr. 59, 1091–1114. 10.1080/10408398.2017.1392289 29040028

[B28] Xiao M. Li Y. Sha L. Gan R. Y. Li H. B. (2018). Natural Products for Prevention and Treatment of Chemical-Induced Liver Injuries. Compr. Revi/ Food Sci. Food Saf. 17, 472–495. 10.1111/1541-4337.12335 33350084

[B29] Yang X. Yang S. Guo Y. Jiao Y. Zhao Y. (2013). Compositional characterisation of soluble apple polysaccharides, and their antioxidant and hepatoprotective effects on acute CCl4-caused liver damage in mice. Food Chem. 138, 1256–1264. 10.1016/j.foodchem.2012.10.030 23411241

[B30] Zeng B. Y. Su M. H. Chen Q. X. Chang Q. Wang W. Li H. H. (2017). Protective effect of a polysaccharide from Anoectochilus roxburghii against carbon tetrachloride-induced acute liver injury in mice. J. Ethnopharmacol 200, 124–135. 10.1016/j.jep.2017.02.018 28229921

[B31] Zhang C. Li J. Wang J. Song X. L. Zhang J. J. Wu S. (2017). Antihyperlipidaemic and hepatoprotective activities of acidic and enzymatic hydrolysis exopolysaccharides from Pleurotus eryngii SI-04. BMC Complement. Alternat. Med. 17, 403. 10.1186/s12906-017-1892-z PMC5557422 28806986

[B32] Zhao K. Xue P. J. Guang-Ye G. U. (2008). Study on Determination of Reducing Sugar Content Using 3,5-Dinitrosalicylic Acid Method. Food Sci. 29, 534–536. 10.3321/j.issn:1002-6630.2008.08.127

[B33] Zhao H. J. Lan Y. F. Liu H. Zhu Y. F. Liu W. R. Zhang J. J. (2017). Antioxidant and Hepatoprotective Activities of Polysaccharides from Spent Mushroom Substrates (Laetiporus sulphureus) in Acute Alcohol-Induced Mice. Oxi. Med. Cell. Long. 2017, 1–12. 10.1155/2017/5863523 PMC5753021 29430281

[B34] Zhou X. Deng Q. F. Chen H. G. Hu E. M. Zhao C. Gong X. J. (2017). Characterizations and hepatoprotective effect of polysaccharides from Mori Fructus in rats with alcoholic-induced liver injury. Int. J. Biol. Macromol. 102, 60–67. 10.1016/j.ijbiomac.2017.03.083 28322946

